# Patient and clinician views on the quality of foot health care for rheumatoid arthritis outpatients: a mixed methods service evaluation

**DOI:** 10.1186/s13047-015-0133-2

**Published:** 2016-01-06

**Authors:** Savia de Souza, Ruth Williams, Heidi Lempp

**Affiliations:** Department of Academic Rheumatology, Faculty of Life Sciences & Medicine, King’s College London, London, UK; Rheumatology Department, King’s College Hospital NHS Foundation Trust, London, UK

**Keywords:** Clinicians, Foot care, Foot health, Mixed methods, Patients, Podiatry, Rheumatoid arthritis, Service evaluation

## Abstract

**Background:**

Feet are often the first site of joint involvement in rheumatoid arthritis (RA) and get progressively worse if unmanaged, leading to permanent disability and negatively impacting patients’ quality of life. Podiatrists are specialists in the assessment, diagnosis and management of foot and ankle problems, however, RA outpatients often rely on referral from rheumatology clinicians to gain access to musculoskeletal podiatry services on the UK National Health Service (NHS). Therefore, the aim of this evaluation was to identify the foot health needs of rheumatoid arthritis patients and if they are being met by rheumatology clinicians.

**Methods:**

A mixed methods approach was used: collecting qualitative data from patients and quantitative data from clinicians. Two focus groups were conducted with nine RA patients from a tertiary rheumatology outpatient clinic in the UK and the data were thematically analysed to inform a clinician survey. Thirteen rheumatology clinicians, from the same centre, completed the online survey. Resultant data were analysed to produce descriptive statistics.

**Results:**

Patient focus group data generated four main themes: (1) need for foot health information, (2) feet ignored during routine consultations, (3) frequency of foot examination and (4) access to podiatry. Survey data highlighted that (i) 69–85 % of clinicians provided patients with foot health information sometimes, (ii) feet were examined in 47 % of routine consultations, (iii) 54 % of clinicians did not examine feet routinely because they are not included in the disease activity score with 28 joints (DAS-28), (iv) 31 % of clinicians referred patients to podiatry upon RA diagnosis, (v) 0 % of clinicians referred patients to podiatry for periodic review, (vi) 54 % of clinicians believed patients will self-report foot problems and (vii) 62 % of clinicians felt competent in foot examination.

**Conclusions:**

RA patients’ foot health needs were not being fully met by rheumatology clinicians. Patients want foot health information and easy access to podiatry services. Rheumatology outpatient consultations need to have a wider focus than the DAS-28 and incorporate foot examination as standard. Clinicians need to ensure they have sufficient training and follow current national foot health guidance to provide optimal foot health care and outcomes for their RA patients.

**Electronic supplementary material:**

The online version of this article (doi:10.1186/s13047-015-0133-2) contains supplementary material, which is available to authorized users.

## Background

Rheumatoid arthritis (RA) is a chronic systemic autoimmune inflammatory disorder seen in adults which primarily affects the bones and cartilage of small and middle sized joints [1]. The foot is the initial site of joint involvement in 36 % of patients with early RA (<2 years) [2]. Up to 57 % of patients report at least mild walking disability within the first year of diagnosis [3]. Synovitis and erosions of the metatarsophalangeal joints occurs early in the RA disease process and if left untreated can lead to severe damage which requires surgery [4].

Pain is the commonest foot symptom [5] and an important predictor of disability [6–8]. Sixty-eight percent of patients with RA have moderate to severe foot pain every day [9]. Foot pain is present even in patients on anti-TNF therapy [10] and when RA is in remission (as classified by the disease activity score with 28 joints (DAS-28)) [11]. Early intervention with foot orthoses improves long-term outcomes by reducing foot pain and disability [12]. Early referral to podiatry is therefore essential.

Foot deformity (as a result of joint damage) can make it difficult for patients to find well-fitting, comfortable shoes [13–15] and may increase the risk of developing painful foot ulcers which pose an additional infection risk to a population reliant on immunosuppressive medication [16]. Foot symptoms negatively affect the quality of life of 94 % of RA patients due to loss of independence, social isolation and depression [17].

In the UK in 2008, the Podiatry Rheumatic Care Association (PRCA) published Standards of Care for People with Musculoskeletal Foot Health Problems [18] and in 2009, The National Institute of Health and Care Excellence (NICE) issued guidelines stating that RA patients should have access to a podiatrist for assessment and periodic review of their foot health needs [19]. Rheumatology clinicians (rheumatologists and clinical nurse specialists) are responsible for coordinating the care of RA patients. Along with general practitioners (GPs), they act as gatekeepers to allied health services such as podiatry.

In some localities, such as the London borough where our tertiary rheumatology outpatient clinic is based, patients can also be referred by allied health professionals or self-refer to podiatry. Walk-in podiatry clinics are available in the community for emergencies such as sudden acute pain, infections and ulcerations. Within our outpatient clinic, rheumatology clinicians can refer patients to our in-house musculoskeletal podiatrist for assessment with any further treatment continued in the community. This would suggest better availability of podiatry services for our RA outpatients compared with other regions in the UK [20].

Foot joint synovitis is predominantly a reflection of systemic disease activity and is present in approximately half of patients at any stage of their RA [21]. However, disease management is largely based around the DAS-28, which omits foot and ankle joints [22], and patients report their feet are ignored [23]. Although clinicians’ views of foot health have been studied alongside those of patients (and parents) in juvenile idiopathic arthritis [24], they have yet to be explored in RA. Therefore, our objective was to identify the foot health needs of RA outpatients and if they are being met by rheumatology clinicians.

## Methods

A mixed methods approach was adopted: collecting qualitative focus group-based data from patients with RA, followed by quantitative survey-based data from clinicians.

### Focus group

Participants were recruited from one tertiary rheumatology outpatient clinic in London (UK) by healthcare staff. Inclusion criteria were adults with established RA, able to read, speak and understand English. The research and development office at King’s College Hospital NHS Foundation Trust reviewed the research proposal in October 2014 and confirmed that ethical committee approval or any further permissions were not required as it is a service evaluation. However, informed written consent was obtained from all patient participants.

Collective views were gathered via two focus groups, which generated rich data about the experiences and beliefs of participants [25]. The focus groups were facilitated by the first author (SdS), who is also a patient with established RA. A topic guide was prepared (see Additional file 1), based on published literature [23, 26, 27] and discussion within the research team, from which questions were asked and further prompts given to facilitate discussion and explore topics in further depth. Focus groups were digitally audio-recorded and transcribed verbatim. Transcripts were verified for authenticity by two randomly selected participants from each focus group and uploaded into the software programme NVivo 10 (QSR International, Doncaster, VC, Australia) for assistance with data analysis.

Data were analysed using inductive thematic analysis within a realist paradigm; whereby analysis was driven by patients’ accounts of their experiences, meaning and reality [28]. Codes were generated by the first author (SdS) and validated by the senior author (HL) to negate potential patient bias. By looking for recurring patterns in the data; themes and sub-themes were identified before finalization of the theme names by authors SdS and HL [29] (see Additional file 2). To enhance validity of the findings; simple counting methods [30] were applied, as well as providing accounts from all participants including negative instances [31].

### Survey

An anonymous online survey was chosen for clinicians to provide easy completion in minimal time and a higher response rate. Questions were developed from themes generated by the focus groups, as well as the researchers’ collective experiences. The survey was piloted and assessed for face and content validity using average congruence [32] from two clinicians (external to the clinic) and our departmental Patient Experts (those living with RA).

The final survey (see Additional file 3) comprised of 11 items in six themes: provision of foot health information, frequency of foot examination, reasons for choosing whether to examine feet, clinician beliefs, podiatry referral and clinician training. Questions encompassed single or multiple responses. Refinements made to the survey based on feedback from the pilot were: 1 question was removed and the wording of 2 questions modified in the ‘clinician beliefs’ section, 1 option was modified in the response set for ‘podiatry referral’ and 2 new options were added to job role for collection of demographic data.

All clinicians from the outpatient clinic were invited, via email by a rheumatology consultant, to fill out the survey online (www.kwiksurveys.com) with one reminder sent 2 weeks later. An information sheet was provided on the first page of the survey and basic demographic data were collected to aid comparative analysis (as variables were highlighted as potential areas of importance by focus group participants). Completion of the survey constituted implied consent. All data were analysed using SPSS 23 (IBM, Armonk, NY, USA) to provide descriptive statistics.

## Results

### Focus group data

Of the twelve patients approached, nine agreed to participate: eight females and one male (7 White, 1 Black, 1 Mixed Race), mean age 50 (range 27–68) years and mean disease duration 16.6 (range 4–46) years. Eight patients experienced current foot and ankle problems that impacted upon their quality of life (see Additional file 4) and two used walking aids. Four main themes were identified from the data across both focus groups (underlined words in patient accounts signify emphasised words):

- **Theme 1: need for foot health information**

There was a general lack of awareness and information about foot health. Some patients knew RA can cause bone erosions and deformities in the feet, but only after experiencing them themselves. Similarly, other patients learned of foot problems, such as arch collapse, bunions, ulcers and persistent verrucae, through personal experience.

Three patients were initially unaware that RA can affect feet and believed it was the clinicians’ role to inform them at diagnosis that they may experience problems with their feet:

*“I think that if they [clinicians] don’t tell you; why would you know that it’s [RA] going to affect your feet; because the first thing you look at, if you look at anything on the internet, you see some hands.”* (Patient 4)

Sources of foot health information seemed limited: two patients looked up information on the internet and one said she would pick up leaflets if she saw them but sometimes these were unavailable. Another patient reported lack of any information:

*“No one gives me any information like how…you have to wear special shoes…I think that is important.”* (Patient 3)

One patient said she did not know what podiatry services were available. As participants seemed to have little knowledge about foot care and services; four resorted to self-manage their foot problems by doing their own exercises, resting their feet up, wearing foam toe caps purchased online or taking off their shoes whenever possible (e.g. on the bus or in the cinema) to get some relief from being in constant pain.

Six patients found provision of information early on in the disease process essential to prevent foot problems. This could either be provided as written information, verbally by a health care professional or delivered in the form of a foot health education group:

*“I think it would be probably quite important as soon as you get a diagnosis to probably start there [feet] because it’s such a load-bearing part of your body that once you have problems with them [feet] I can imagine that it’s…such a big issue…So I think it would be good to start off by…maybe sending you to a podiatrist or a group who will…talk about ‘ok, you need to wear flat shoes, you need to circle your feet and take care of them’…”* (Patient 9)

Overall, patients wanted information through the rheumatology outpatient clinic on foot care, footwear, what local foot health services are available and when and how to seek help:

*“The [rheumatology] department, they have to think of…how you can buy shoes, how you can maintain the wellbeing of your feet…They have to give you some kind of guidance.”* (Patient 7)

One patient (who also had diabetes) drew a comparison between the quality of foot care in RA and the care she received when first diagnosed with diabetes:

*“I think that’s where diabetic foot care is much better, because you are taught to examine your feet and what to look for and where and how to examine. You get a lot of written information when you’re first diagnosed with diabetes as well, about foot care. So the same thing should really apply to rheumatology.”* (Patient 2)

Adequate provision of information early on in the RA disease process was seen as key to preventing foot problems developing or worsening. Patients stated that once foot problems started, deterioration could rapidly ensue, with one patient expressing despair that no ‘solution’ had been found for her feet.

- **Theme 2: feet ignored during routine consultations**

Eight out of nine patients stressed that feet were ignored by rheumatology clinicians during routine consultations:

*“I think sometimes they ask…if any of your joints hurt, and you say ‘my feet’, but then it just seems to not really be acknowledged.”* (Patient 8)

*“I think that when you go to the clinic; they don’t really concern themselves with your feet do they? They don’t really ask how your toes are or whether you’re having any pain there?…They don’t ask to look at ‘em [feet] at all.”* (Patient 5)

Five patients linked foot problems to RA disease activity and were surprised that feet are not included in the DAS-28 (which clinicians routinely use to assess how active RA is):

“So you can have, which I did have, at least 8 swollen joints in my feet and they do not come into the joint count!…It’s almost like saying that they [feet] are not part of your body and I don’t understand that.” (Patient 6)

It seemed feet were given little importance: six patients commented that emphasis was placed on hands and feet ‘get left behind’ [Patient 6]. Three patients emphasised that feet may be more affected by RA than hands, and if ignored could lead to inadequate disease management and avoidable problems:

*“I think the feet tell consultants a lot more…If you take me for example…my hands are not bad…whereas my feet are what most people would expect my hands to be. So if they kept track of my feet, I probably wouldn’t have what I’ve got now with this second toe, that’s gone like that [deformed].”* (Patient 4)

Feet were not routinely examined. Seven patients reported that clinicians only look at their feet if they raise the subject themselves. However, two patients found their feet ignored even when they brought up their concerns, which led to feelings of annoyance at not being listened to:

*“I was in a rheumatoid appointment with the nurse and I said to her…about my feet, but she only looked at my hands and did the [joint] count on my hands…A week later, my…foot…went huge. I was due for my infusion and…a doctor…said ‘do you realise you’re having a flare?’ and she [rheumatology nurse]…[had] totally discounted what I was telling her about my feet.”* (Patient 2)

There was reluctance by some patients to raise foot problems with their rheumatology healthcare team. Three patients were so used to living with chronic pain that they only ever raised foot problems if they caused a lot of pain whilst another was concerned about seeming a nuisance:

*“Well you get used to pain don’t you? That’s the trouble and if it’s an average pain then you’ll probably always think that’s fine.”* (Patient 1)

*“Last week I had a bad flare up in me ankles and they just swelled up…but I didn’t know what to do about it. So what I done was go to bed and put me feet up on some pillows…The thing is like today [at clinic appointment] they were saying ‘why didn’t you ring us up?’. I say ‘Cos I don’t like to bother you’.”* (Patient 5)

One patient suspected that limited time for consultations and a lack of training may be responsible for clinicians not routinely examining feet:

*“No one ever asks you really about your feet or looks at your feet because it takes too long, well that’s what I always think in clinic…I think GPs and medical schools, everything concentrates on hands. Although they perhaps say…‘hands and feet are small joints that are where it [RA] most commonly presents’…So maybe they [clinicians] lack confidence in examining feet?”* (Patient 8)

- **Theme 3: frequency of foot examination**

Almost all participants stated it was very important that feet are always examined. Eight patients wanted foot examination to be a standard, recorded part of routine consultations so that the information can be considered along with the DAS-28 and blood results when planning treatment:

*“I think that…because it’s [about feet] not part of the assessment, it doesn’t get asked. I think it needs to be included in the [routine] assessment and then it’s like a tick box (cos you do feel sometimes it’s a tick box [exercise]). It’s [feet] just a fundamental thing…”* (Patient 2)

*“It’s got to be on that chart so when they’re looking at it: they’re reading your bloods, your DAS[−28] score and your feet score. The whole lot’s there together.”* (Patient 4)

Conversely, one patient explained that she did not mind if not asked specifically about her feet during consultations:

*“I’ve had a different experience when I go to the rheumatologist…In the past they always would…check my feet…Nowadays they tend to just ask me, because I haven’t had problems with my feet for…5 years…So they don’t specifically ask about that. I have problems with my hands that they’ll ask specifically about…but they’ll ask me ‘Does anything else hurt? Are there any problems with anything?’. So it…gives me the opportunity to pick up on anything.”* (Patient 9)

- **Theme 4: access to podiatry**

All participants were in agreement that patients need to be referred to a podiatrist for assessment immediately after being diagnosed with RA as a matter of course to prevent foot problems occurring in the future. Again, comparisons were drawn with diabetic foot care:

*“You’re seen earlier in diabetes…when someone’s not really likely to have any foot complications so why not do the same with rheumatoid?”* (Patient 8)

Six patients reported they should have the right to access podiatry whenever needed due to the chronic and unpredictable nature of RA:

*“You’re going to have a problem aren’t you? If you’ve got rheumatoid arthritis…there are going to be issues with your feet.”* (Patient 4)

*“I think once you got rheumatoid, you got a case for going to podiatry. So I think it would be easy; the door’s… always open for ya.”* (Patient 5)

Interestingly, patients mentioned they were more likely to be referred to podiatry services by a rheumatology clinical nurse specialist or their GP, than by a rheumatologist.

Four patients (mostly older participants) experienced difficulty with looking after their own feet due to problems with other joints and therefore demanded regular basic foot care to be provided for them on the National Health Service (NHS) as it is for diabetic patients:

*“I think…certainly for me as I get older…,and I’ve had a hip replaced…, reaching your feet…becomes a real problem. Just basic foot care like cutting [toe]nails, cos your hands aren’t great you can’t do it [cut toenails] with scissors. So shouldn’t we get chiropody on the NHS like diabetic patients?”* (Patient 8)

Due to lack of regular access to podiatry on the NHS, one patient frequently visited a private podiatrist for basic foot care:

*“Well I have to. We are all chronic [RA and diabetes patients] and we are all dependent on our feet totally regardless of whether they flare up or don’t, and they hurt every time I walk…”* (Patient 1)

All patients seemed unaware self-referral to community podiatry was possible within the local area but given this option, three patients expressed a preference to be referred through the rheumatology clinic so their doctor would be aware they were experiencing foot problems:

*“The…thing about self-referral is…I’m not sure about how well the podiatrist connects with the rheumatology department. That’s why I do it [get referred] through the [rheumatology nurses’] helpline.”* (Patient 6)

### Survey data

13/18 rheumatology clinicians approached took part (response rate = 72 %): 5 consultants, 4 specialist registrars, 1 clinical fellow and 3 clinical nurse specialists (5 males:8 females). 7 clinicians had completed their undergraduate training in the UK and 6 abroad. 10 had completed the majority of their postgraduate training in the UK, 1 abroad and 2 were still in training.

- **Provision of foot health information**

See Table 1. Cross-referencing with demographic data found no trends by role or gender.

**Table 1:** Frequency of information being provided to patients

*I provide RA patients with…*	Never	Only at diagnosis	Sometimes	Always
information on how RA can affect feet	0.0 %	7.7 %	84.6 %	7.7 %
foot care advice	7.7 %	--	84.6 %	7.7 %
footwear advice	15.4 %	--	69.2 %	15.4 %
information on when and how to access local podiatry services	7.7 %	--	84.6 %	7.7 %

- **Frequency of foot examination**

**Fig. 1:**
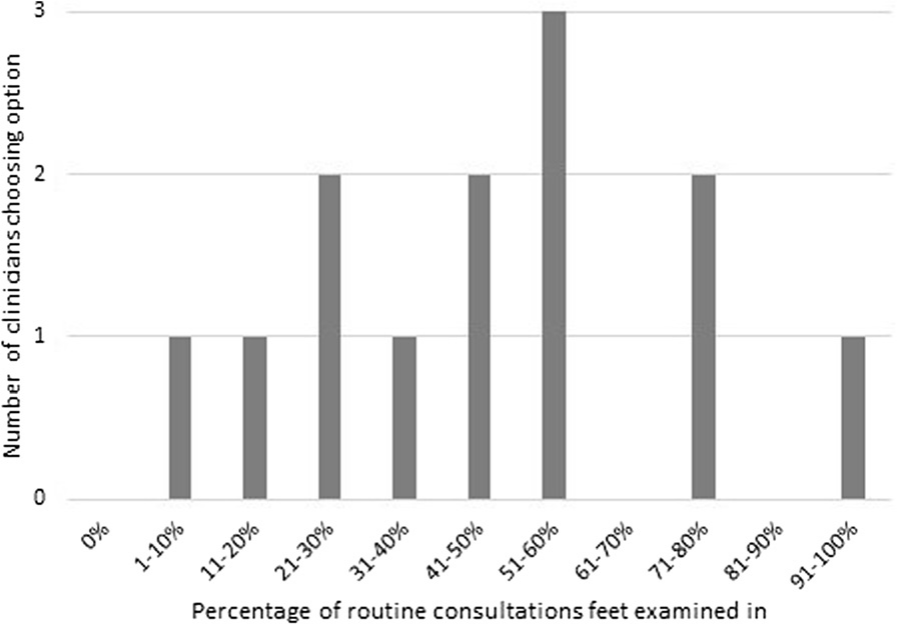
Percentage of routine consultations feet examined in (single response)

The data showed a wide distribution (Fig. 1) with an overall mean of 47 %. By cross-referencing with demographic data, it appeared that female consultants were more likely to examine feet regularly (range 51–80 %), followed by female specialist registrars (range 41–80 %), male specialist registrars (range 51–60 %), clinical nurse specialists [all female] (range 11–50 %) and male consultants (range 1–30 %). (As only one clinical fellow participated in the survey, their data were not included in this subsection of analysis.)

- **Reasons for choosing whether to examine feet**

**Fig. 2:**
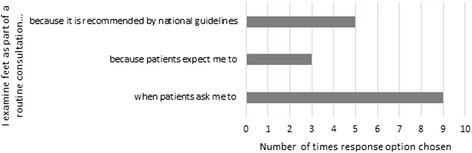
Reasons for examining feet (multiple response)

**Fig. 3:**
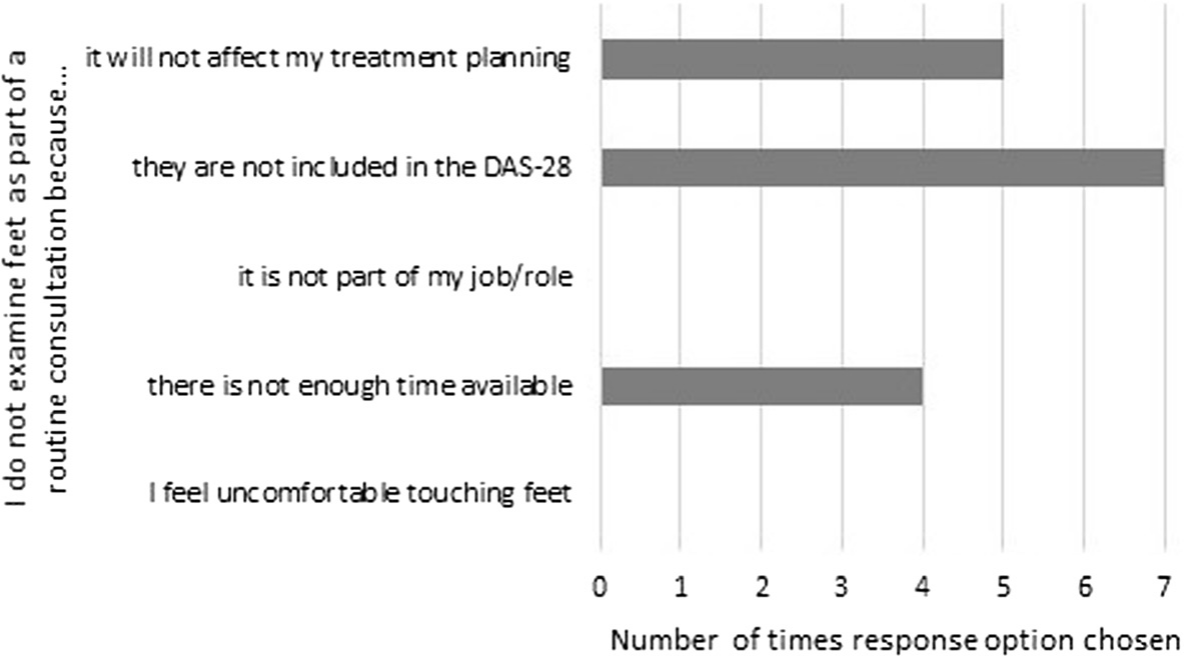
Reasons for not examining feet (multiple response)

See Figs. 2 and 3. A cross-check with demographic data showed that clinical nurse specialists only examined feet when asked by patients. Job role or gender did not affect response choice for not examining feet.

- **Clinician beliefs**

See Table 2. Cross-referencing demographic data showed that 80 % (4/5) of consultants strongly agreed that RA patients often have foot problems, 100 % (3/3) of clinical nurse specialists agreed and the response from specialist registrars was spread across neutral to strongly agree. Eighty percent (4/5) of consultants strongly agreed that foot problems are an important indicator of RA disease activity. One hundred percent of registrars (4/4) and nurses (3/3) agreed. Opinion was divided amongst the clinician groups on whether patients will self-report foot problems. Sixty-two percent (8/13) of clinicians (including all consultants) felt competent in foot examination. There was no response-bias based on gender for any of the statements.

**Table 2:** Clinician belief statements

	Strongly Disagree	Disagree	Neutral	Agree	Strongly Agree	Average Score
Patients often have foot problems as a result of having RA	0/13 (0.0 %)	0/13 (0.0 %)	1/13 (7.7 %)	6/13 (46.2 %)	6/13 (46.2 %)	4.4/5
Foot problems are an important indicator of RA disease activity	0/13 (0.0 %)	0/13 (0.0 %)	1/13 (7.7 %)	8/13 (61.5 %)	4/13 (30.8 %)	4.2/5
Patients will tell me if they have problems with their feet	0/13 (0.0 %)	3/13 (23.1 %)	3/13 (23.1 %)	4/13 (30.8 %)	3/13 (23.1 %)	3.5/5
I feel competent examining feet	1/13 (7.7 %)	1/13 (7.7 %)	3/13 (23.1 %)	7/13 (53.9 %)	1/13 (7.7 %)	3.5/5

- **Podiatry referral**

**Fig. 4:**
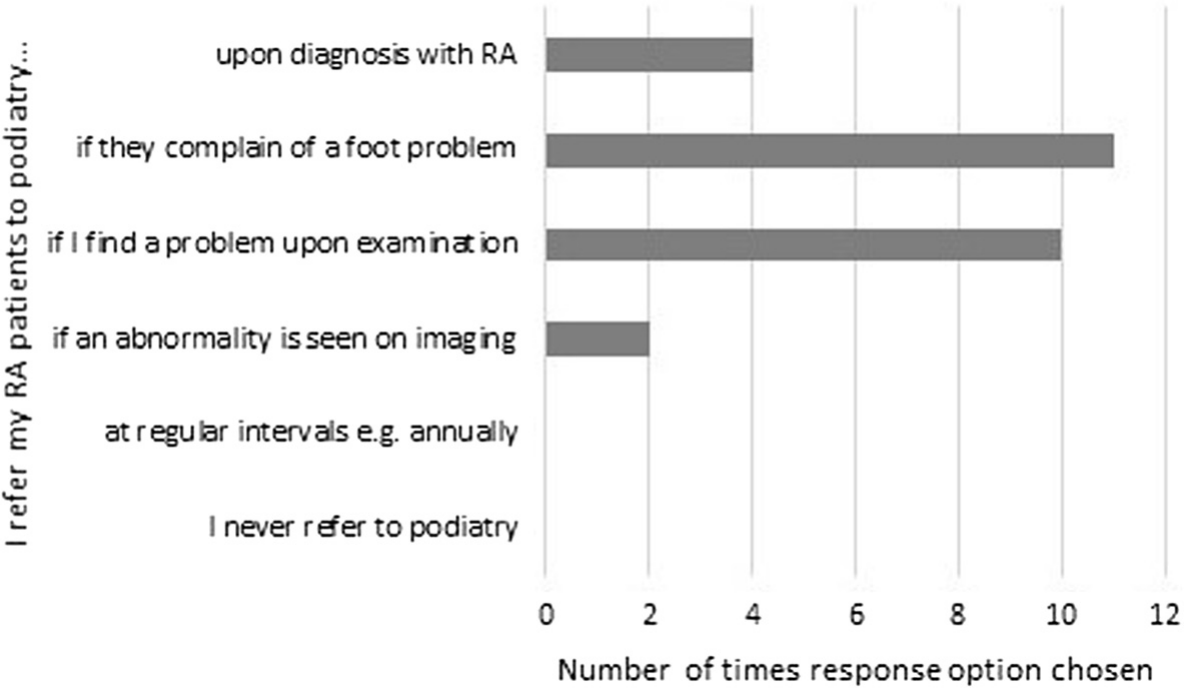
When patients are referred to podiatry (multiple response)

See Fig. 4. A cross-check with demographic data showed no difference in response by role or gender.

- **Clinician training**

During undergraduate training, 0 % (0/5) of consultants, 25 % (1/4) of registrars and 33 % (1/3) of nurse specialists examined rheumatoid feet. During postgraduate rheumatology training, 80 % (4/5) of consultants, 75 % (3/4) of registrars and 67 % (2/3) of nurses examined feet. (Clinical Fellow data were excluded as only one respondent.) Cross-referencing the results showed that where clinicians had trained as undergraduates or postgraduates (i.e. in the UK or abroad) seemed to have no bearing on their likelihood to examine feet.

## Discussion

This evaluation is the first to systematically study the views of patients and clinicians on the quality of foot health care in RA in one outpatient clinic. The results indicate that RA patients’ foot health needs were not being fully met by rheumatology clinicians. Provision of foot health information by clinicians seemed to be inconsistent, in line with recent publications [26, 27, 33, 34]. Although charities Arthritis Research UK and the National Rheumatoid Arthritis Society provide comprehensive patient foot health information [35, 36], we found patients to be unaware of these resources.

Patients appeared to be unclear when and how to seek help and some resorted to uninformed self-management. This was contrary to PRCA Standards of Care, which state that patients should be supported to self-manage through information provision, know when to seek help and how to access relevant services [18]. Patients were unaware they could self-refer to community podiatry but some expressed a preference to be referred to our in-house podiatrist by the rheumatology clinic.

In light of these findings, our departmental podiatrist produced a foot health information leaflet for all outpatient clinic attendees that describes how RA can affect feet, what can be done to help and how best to be referred to podiatry (see Additional file 5). This leaflet was approved by our departmental Patient Experts and is now available in the outpatient clinic waiting room and given to newly diagnosed patients.

Clinician data showed that no patients were referred to podiatry periodically as per NICE guidelines [19]. Foot examination during routine consultations was found to be highly variable, despite nearly all clinicians believing foot problems are an important indicator of RA disease activity, and emerged as a higher priority in female than in male doctors (particularly amongst consultants). Future research into this possible gender effect would be valuable.

Patients wanted foot examination to be a standard, recorded part of routine consultations. The dominant reason clinicians gave for not examining feet was that they are not included in the DAS-28. That consultations were more driven by the DAS-28, with a greater focus on hand than foot examination, is of concern [9, 23] as persistent active synovitis can be present in foot joints of patients classified as being in remission by the DAS-28 [11, 22, 37].

It has recently been suggested that the 66/68-joint count [38] be used routinely to assess patients with psoriatic arthritis, as it includes feet and ankles [39]. This could also be applied for RA, however, it is time-consuming and inter-examiner variability is higher than for the 28-joint count [40]. Alternatively, an RA-specific foot patient-reported outcome measure, such as the Leeds Foot Impact Scale [41] or the Salford Arthritis Foot Evaluation instrument [42], could be employed as a useful adjunct to current clinical practice.

Interestingly, over half of clinicians relied on patients to raise foot problems. However, patients do not always report symptoms as they do not want to appear as a nuisance to their healthcare team, believe a certain amount of pain is to be expected/endured or are in denial that their disease may be worsening [26]. Therefore, it is important for clinicians to assess feet during routine consultations to identify problems that may otherwise be left untreated. The survey revealed that not all clinicians felt competent in foot examination, due to receiving little training in the rheumatoid foot to enable them to effectively assess and manage foot problems [43, 44]. Thus, further postgraduate training seems to be required in this area.

All patients viewed a referral to podiatry immediately after RA diagnosis as essential to prevent permanent foot damage. PRCA Standards recommend that all patients receive a foot health assessment within 3 months of RA diagnosis [18]. However, in line with the Rheumatology Futures Group Report (2009) [45], we found that only approximately one-third of clinicians adhered to this recommendation.

All patients requested easy access to NHS podiatry services whenever required and some expressed the opinion that a comprehensive foot health service provision, similar to that for diabetic patients, is needed. This question has been previously raised [43, 44]. However, whilst outcomes for diabetic foot ulcers have improved with early referral to podiatry, comprehensive diabetic foot care comes at a substantial cost to the NHS [46].

Despite there being good foot health service provision in our local area; access for RA outpatients seemed limited due to lack of awareness by patients and rheumatology clinicians alike. It is apparent from our findings that problems exist within the service level design. We therefore intend to carry out further service development (in conjunction with patients, rheumatology clinicians and podiatrists) on how best to raise awareness of foot health and reconfigure services.

### Strengths and limitations

A particular strength of this evaluation was that the clinician response rate was above average [47] at 72 %. This is likely due to the survey invite email and reminder being sent out to clinic staff individually by a consultant and the survey being anonymous (thus, more likely to produce honest answers).

Limitations of the evaluation include a non-validated questionnaire being developed to obtain retrospective views from clinicians (as no validated psychometric questionnaire was available for this particular purpose), the potential for recall bias [48] in both patient accounts and clinician responses and the potential for response bias in questionnaire answer selection. Efforts were made to limit response bias by programming the online survey to apply randomisation and rotation of answers where appropriate [49]. The evaluation is also limited as it represents a single tertiary outpatient clinic. Thus, the results are not generalizable.

However, many of the patient views were reflective of previous studies [14, 23, 27, 33], thus validating our findings. It would have been helpful to have included free text boxes in the survey to gather some qualitative data. In-depth face-to-face interviews with clinicians to further explore issues raised by the survey would be important for future research.

## Conclusions

This service evaluation provides a snapshot of current clinical practice at a local level in regards to the quality of foot health care for RA outpatients in the UK. Along with previous papers [33, 50, 51], it supports the need for national guidance [18, 19] to be implemented in rheumatology clinics to improve long-term foot health outcomes for RA patients. More work needs to be done to raise the profile of foot health amongst rheumatology clinicians and patients. Clinicians need to take into account foot symptoms when managing patients and avoid so-called ‘DAS blindness’ (where the consultation focuses purely on components pertinent to the DAS-28) [52]. Rheumatology clinic staff may require further postgraduate training in foot examination and need to include podiatry as part of standard multidisciplinary care for RA patients.

### Availability of supporting data

The data sets supporting the results of this article are included within the article and its additional files.

## Additional files

Additional file 1:
**Focus group topic guide.** (DOCX 17 kb) Additional file 2:
**Thematic analysis breakdown.** (DOCX 14 kb) Additional file 3:
**Clinician survey (paper format).** (PDF 296 kb) Additional file 4:
**Problems currently experienced by focus group patients.** (DOCX 12 kb) Additional file 5:
**Foot health patient information leaflet.** (PDF 443 kb)
