# Supplementary material for: Patient and clinician views on the quality of foot health care for rheumatoid arthritis outpatients: a mixed methods service evaluation
Source: J Foot Ankle Res. 2016 Jan 6;9:1. doi: 10.1186/s13047-015-0133-2 (PMC4702354; doi:10.1186/s13047-015-0133-2)
Supplement: Additional file 1: — Focus group topic guide. (DOCX 17 kb) [file 13047_2015_133_MOESM1_ESM.docx]

**Focus group topic guide**

1. What do you know, in general, about how feet can be affected by rheumatoid arthritis?

- What are your sources of information?

1. What problems do you have with your feet because of your rheumatoid arthritis?

- Does it affect you doing your normal activities?
- Do you get any help with foot problems? From whom?

1. Does your rheumatologist/rheumatology nurse ask about your feet during consultations?

- What advice and treatment would be helpful to you from the rheumatology team?
- What information do you need?

1. How easy is it for you to get referred to a podiatrist?

- Have you ever self-referred to a podiatrist?
