# Supplementary material for: Patient and clinician views on the quality of foot health care for rheumatoid arthritis outpatients: a mixed methods service evaluation
Source: J Foot Ankle Res. 2016 Jan 6;9:1. doi: 10.1186/s13047-015-0133-2 (PMC4702354; doi:10.1186/s13047-015-0133-2)
Supplement: Additional file 2: — Thematic analysis breakdown. (DOCX 14 kb) [file 13047_2015_133_MOESM2_ESM.docx]

**Thematic analysis breakdown**

| **Subtheme** | **Number of references in transcripts** | **Main theme** |
| --- | --- | --- |
| - Lack of information from rheumatology department | 18 | Need for foot health information |
| - Want information from the rheumatology department | 13 |  |
| - Prevention | 15 |  |
| - Want footwear advice | 8 |  |
| - Patients having to self-manage | 7 |  |
| - Feet neglected by clinicians | 44 | Feet ignored during |
| - Emphasis on hands | 17 | routine consultations |
| - Want regular foot care service for rheumatology patients | 9 |  |
| - Doctors’ training - Annoyance | 9  7 |  |
| - Feet linked to RA disease activity | 6 |  |
| - Feet only looked at if raised by patient | 5 |  |
| - Feet not looked at when raised | 5 |  |
| - Patients reluctant to raise foot problems | 3 |  |
| - Want attention given to feet during rheumatology consultations | 15 | Frequency of foot examination |
| - Does not mind attention not being given to feet specifically | 1 |  |
| - Referral to podiatrist experience | 22 | Access to podiatry |
| - Want easy access to podiatry | 13 |  |
| - Difficulty looking after own feet | 7 |  |
| - Having to see a private podiatrist | 4 |  |
| - Patients unaware they can self-refer - Patients unaware they can ask for a podiatry referral - Patients not keen to self-refer | 4  2  1 |  |
