# Supplementary material for: Patient and clinician views on the quality of foot health care for rheumatoid arthritis outpatients: a mixed methods service evaluation
Source: J Foot Ankle Res. 2016 Jan 6;9:1. doi: 10.1186/s13047-015-0133-2 (PMC4702354; doi:10.1186/s13047-015-0133-2)
Supplement: Additional file 3: — Clinician survey (paper format). (PDF 296 kb) [file 13047_2015_133_MOESM3_ESM.pdf]

Clinicians' views on foot health in rheumatoid arthritis (RA)

We would like to invite you to participate in this **service evaluation**. Participation is voluntary and should take about **5 minutes** to complete. We are interested in how you view and treat foot problems in your patients with rheumatoid arthritis. This survey is **anonymous** and is part of a project to **help improve outpatient services** here at xxx.

Submission of a completed questionnaire implies consent to participate (by pressing 'Finish Survey'). Partially completed questionnaires will not be analysed. As well as helping improve rheumatology outpatient services, the findings from the data collected may be published in journals or disseminated at conferences.

If you have any questions or require more information about this evaluation, please contact the researcher using the following contact details: Dr Savia de Souza, Academic Department of Rheumatology, 3rd Floor Weston Education Centre, London, SE5 9RJ (savia.de_souza@kcl.ac.uk). Or alternatively, you can contact the research supervisor: Dr Heidi Lempp (heidi.lempp@kcl.ac.uk).

Provision of foot health information

1. I provide patients with information on how RA can affect feet

- ○ Always
- ○ Sometimes
- ○ Never
- ○ Only at diagnosis

2. I provide RA patients with foot care advice

- ○ Always
- ○ Sometimes
- ○ Never

3. I provide RA patients with footwear advice

- ○ Always
- ○ Sometimes
- ○ Never

4. **I provide RA patients with information on when and how to access local podiatry services**

- ○ Always
- ○ Sometimes
- ○ Never

Frequency of foot examination

5. **Thinking ONLY of your patients with** RA**...in what percentage (roughly) of *routine* consultations do you examine feet?**

- ○ 0%
- ○ 1-10%
- ○ 11-20%
- ○ 21-30%
- ○ 31-40%
- ○ 41-50%
- ○ 51-60%
- ○ 61-70%
- ○ 71-80%
- ○ 81-90%
- ○ 91-100%

Reasons for choosing whether to examine feet

6. **I examine feet as part of a *routine* consultation…(tick *ALL* which apply)**

- ○ When patients ask me to
- ○ Because patients expect me to
- ○ Because it is recommended by national guidelines

7. **I *do not* examine feet as part of a *routine* consultation because …(tick *ALL* which apply)**

- ○ It will not affect my treatment planning
- ○ They are not included in the DAS-28
- ○ It is not part of my job/role
- ○ There is not enough time available
- ○ I feel uncomfortable touching feet

Clinician beliefs

8. **Please choose the option closest to your views for the following statements:**

	Stongly disagree	Disagree	Neutral	Agree	Strongly agree
Patients often have foot problems as a result of having RA	○	○	○	○	○
Foot problems are an important indicator of RA disease activity	○	○	○	○	○
Patients will tell me if they have problems with their feet	○	○	○	○	○
I feel competent examining feet	○	○	○	○	○

Podiatry referral

9. **I refer my RA patients to podiatry …(tick *ALL* which apply)**

- ○ Upon diagnosis with RA
- ○ If they complain of a foot problem
- ○ If I find a problem upon examination
- ○ If an abnormality is seen on imaging
- ○ At regular intervals, e.g. annually, even if the patient has no foot complaints
- ○ I never refer to podiatry

Clinician training

10. When presented with an RA patient during *undergraduate* training I examined their…

- ○ Hands only
- ○ Hands and feet
- ○ Feet only
- ○ Not applicable

11. When presented with an RA patient during *postgraduate rheumatology* training I examined their…

- ○ Hands only
- ○ Hands and feet
- ○ Feet only

Demographic data

12. I am a…

- ○ Consultant
- ○ Specialist registrar
- ○ Specialist nurse
- ○ Clinical fellow
- ○ Trainee – other

The last 3 questions are *optional* but will greatly assist with comparative analysis.

13. I am…

- ○ Male
- ○ Female

14. I completed my *undergraduate* training in…

- ○ UK
- ○ Abroad

15. I completed the majority of my *postgraduate rheumatology* training in…

- ○ UK
- ○ Abroad

Please don't forget to click 'Finish Survey'.

Thank you for completing the survey.
