# Supplementary material for: Patient and clinician views on the quality of foot health care for rheumatoid arthritis outpatients: a mixed methods service evaluation
Source: J Foot Ankle Res. 2016 Jan 6;9:1. doi: 10.1186/s13047-015-0133-2 (PMC4702354; doi:10.1186/s13047-015-0133-2)
Supplement: Additional file 4: — Problems currently experienced by focus group patients. (DOCX 12 kb) [file 13047_2015_133_MOESM4_ESM.docx]

**Foot and ankle problems currently experienced by focus group patients**

- Ankles – pain and swelling
- Feet have spread/got wider
- Curled/crooked toes
- Pain in metatarsophalangeal joints
- Bunions
- Vasculitis
- Chilblains
- Ulcers
- Persistent verrucae – won’t go due to immunosuppressive medication
- Inflammation and swelling
- Pain/discomfort with touching feet, standing, walking and at rest
