# Supplementary material for: Patient and clinician views on the quality of foot health care for rheumatoid arthritis outpatients: a mixed methods service evaluation
Source: J Foot Ankle Res. 2016 Jan 6;9:1. doi: 10.1186/s13047-015-0133-2 (PMC4702354; doi:10.1186/s13047-015-0133-2)
Supplement: Additional file 5: — Foot health patient information leaflet. (PDF 443 kb) [file 13047_2015_133_MOESM5_ESM.pdf]

The Rheumatology Foot Clinic is provided by a team of podiatrists. Dedicated sessions at Kings Rheumatology take place on Monday and Friday afternoons.

Ask any member of the Rheumatology team to make a referral to us.

If you come to the clinic for an appointment:

Foot problems can be caused by forces acting from higher in the body. We may therefore need to examine you from the spine downwards.

- Please wear clothing that allows us to see your legs from the knees down, and avoid using cream on your legs that day.
- Bring your usual shoes, trainers if you have them, any shoes required for special activities and any current or previous insoles or shoes that have been prescribed for you or you have bought.

Confidentiality:

All information we take from you is stored securely on a computer system. You can request to see it at any time.

Students:

The department take students on placement. If they are present during your consultation they will be supervised. *Please let us know when you come to the clinic if you would prefer a student not to be present*.

There is high demand for this service so please do not miss any appointments and attend on time. Please ensure we have correct contact details in case we need to rearrange appointments.

Rheumatology Foot Clinic

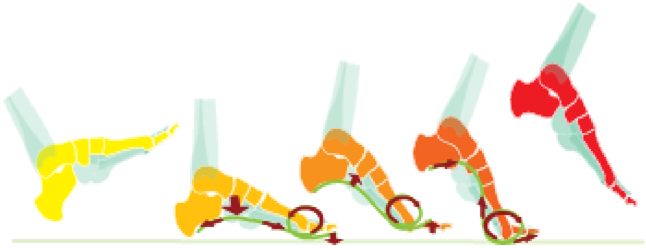

If you have rheumatoid arthritis:

- Do you have any current foot pain?
- Do you find it difficult to find shoes to fit?
- Do you have numbness in your legs or feet?
- Are your feet excessively cold or do you get cramp on walking?
- Do you have any painful corns or calluses?
- Do you have any foot wounds?
- Do you have any problems with your toenails?

If the answer is no, then keep this leaflet to refer to in case you develop any problems in the future.

If the answer is yes to any of these questions, then read on for further information and ask your Rheumatology nurse or consultant for a referral to a Podiatrist at King's Rheumatology Foot Clinic .

Rheumatoid arthritis? what can the Rheumatology Foot Clinic do for you?

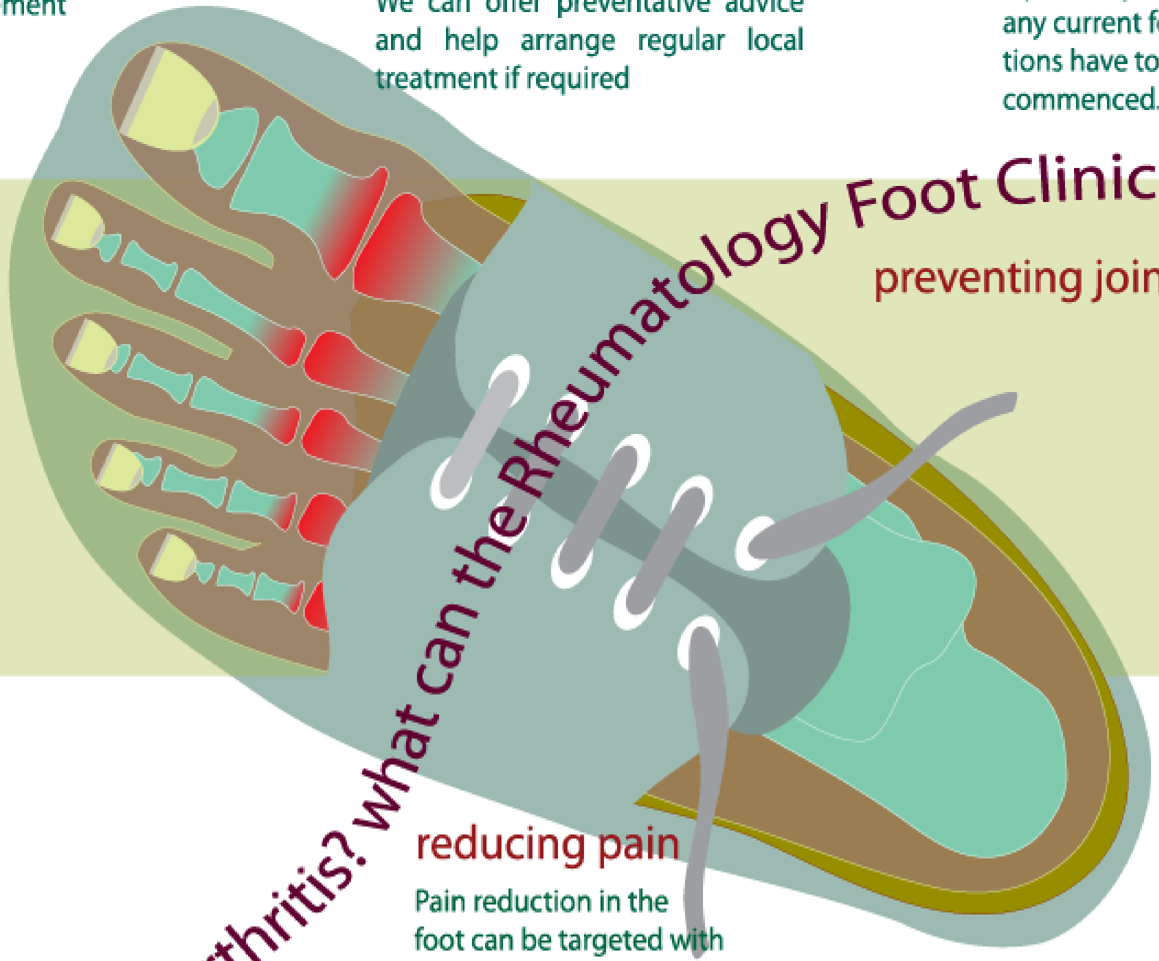

nail problems

Toenails can become thickened, infected with fungus or may 'in-grow' at the sides.
We can advise on management and treat as required.

corns & calluses

Changes in foot shape with arthritis can cause pressure points on the toes and under the ball of the foot. We can offer preventative advice and help arrange regular local treatment if required

infections

Medication for arthritis can affect your immune system so infections may not heal so readily.
If you are planning Anti TNF treatment, any current foot wounds or skin infections have to heal before treatment is commenced. We can help with this.

circulation and sensation

Arthritis can affect circulation and sensation. We undertake checks on this, so if you have been noticing calf cramps on walking or numbess then ask for assessment

preventing joint pain & deformity

~foot orthoses

Early treatment with specialised insoles to hold the foot in a good alignment has been shown to reduce pain and deformity in the longer term. Come and see us for an individualised assesment

~footwear

Shoes can significantly help or hinder the way the foot works. See the us for individulaised advice that lasts a lifetime

onward referral

Sometimes we may need to use the skills of others, such as Physiotherapy, Orthotists and foot Surgeons

reducing pain

Pain reduction in the foot can be targeted with laser treatment, acupuncture and steroid injections used as part of a treatment plan
